# 帕博利珠单抗治疗晚期非小细胞肺癌安全性和有效性的真实世界研究

**DOI:** 10.3779/j.issn.1009-3419.2024.106.29

**Published:** 2024-10-20

**Authors:** Ning WAN, Bing WANG, Ya GUO, Zijian HE, Chen YANG, Ning YANG, Liqing LU, Hongyi LIANG, Weibin XIAO, Dandan YANG, Zhuojia CHEN, Wenfeng FANG, Weiting LIANG

**Affiliations:** ^1^510010 广州，中国人民解放军南部战区总医院临床药学科; ^1^Department of Clinical Pharmacy, General Hospital of Southern Theater Command, Guangzhou 510010, China; ^2^510515 广州，南方医科大学药学院; ^2^School of Pharmaceutical Sciences, Southern Medical University, Guangzhou 510515, China; ^3^510006 广州，广州中医药大学药学院; ^3^Department of Pharmacy, Guangzhou University of Chinese Medicine, Guangzhou 510006, China; ^4^528306 佛山，佛山市顺德区和祐医院药学部; ^4^Department of Pharmacy, Heyou Hospital, Shunde District, Foshan 528306, China; ^5^510623 广州，暨南大学药学院; ^5^College of Pharmacy, Jinan University, Guangzhou 510623, China; ^6^510010 广州，中国人民解放军南部战区总医院肿瘤科; ^6^Department of Oncology, General Hospital of Southern Theater Command, Guangzhou 510010, China; ^7^510120 广州，中山大学孙逸仙纪念医院药学部; ^7^School of Pharmaceutical Sciences, Sun Yat-sen University, Guangzhou 510120, China; ^8^510060 广州，中山大学肿瘤防治中心药学部，华南恶性肿瘤防治全国重点实验室，广东省恶性肿瘤临床医学研究中心; ^8^Department of Pharmacy, Sun Yat-sen University Cancer Center, State Key Laboratory of Oncology in South China, Guangdong Provincial Clinical Research Center for Cancer, Guangzhou 510060, China; ^9^510060 广州，中山大学肿瘤防治中心内科，华南恶性肿瘤防治全国重点实验室，广东省恶性肿瘤临床医学研究中心; ^9^Department of Medical Oncology, Sun Yat-sen University Cancer Center, State Key Laboratory of Oncology in South China, Guangdong Provincial Clinical Research Center for Cancer, Guangzhou 510060, China

**Keywords:** 真实世界数据, 帕博利珠单抗, 肺肿瘤, 安全性, 有效性, Real-world data, Pembrolizumab, Lung neoplasms, Safety, Efficacy

## Abstract

**背景与目的:**

帕博利珠单抗（Pembrolizumab, PEM）治疗晚期非小细胞肺癌（non-small cell lung cancer, NSCLC）在临床试验中被证实有效，但这些试验是基于按照特定标准筛选的患者群体，因此这些结果是否能够代表真实世界中患者的普遍情况，仍然值得讨论。本研究旨在基于真实世界数据评估PEM治疗晚期NSCLC的有效性和安全性。

**方法:**

回顾性收集接受PEM治疗的晚期NSCLC患者的真实世界数据，使用倾向性评分匹配消除组间差异，评估PEM与化疗的有效性和安全性。

**结果:**

在倾向性评分匹配后的450例患者中PEM组和化疗组任何等级不良事件发生率分别为79.87%和86.71%，≥3级不良事件发生率分别为4.03%和7.31%。PEM组和化疗组的客观缓解率分别为48.63%和36.00%（P=0.011），中位无进展生存期分别为15.5和8.8个月（P<0.001），中位总生存期分别为未达到和26.2个月（P<0.001）。

**结论:**

PEM治疗晚期NSCLC在实际临床应用中显示出较好的生存率和可接受的安全性。

## Abstract (en)

**Background and objective:**

Pembrolizumab (PEM) has been shown to be effective in clinical trials for the treatment of advanced non-small cell lung cancer (NSCLC), but clinical trials were based on cohorts of patients selected on specific criteria, and whether the findings are consistent with real-world patients is debatable. The aim of this study is to evaluate the efficacy and safety of PEM in the treatment of advanced NSCLC based on real-world data.

**Methods:**

A retrospective collection of real-world data from patients with advanced NSCLC receiving PEM was conducted. Propensity score matching was used to eliminate inter-group differences and assess the efficacy and safety of PEM compared to chemotherapy.

**Results:**

Among 450 matched patients, the incidence rates of any-grade adverse events were 79.87% in the PEM group and 86.71% in the chemotherapy group, while the incidence rates of grade ≥3 adverse events were 4.03% and 7.31%, respectively. The objective response rates were 48.63% for PEM and 36.00% for chemotherapy (P=0.011). The median progression-free survival was 15.5 months for PEM and 8.8 months for chemotherapy (P<0.001), and the median overall survival was not reached for PEM and 26.2 months for chemotherapy (P<0.001).

**Conclusion:**

PEM treatment for advanced NSCLC demonstrates favorable survival outcomes and acceptable safety in real-world clinical practice.

在我国肺癌的发病率和死亡率均位居首位，其中非小细胞肺癌（non-small cell lung cancer, NSCLC）占比80%-85%，主要包括腺癌和鳞状细胞癌等类型，就诊时至少70%患者为晚期^[1⇓-3]^。对于表皮生长因子受体（epidermal growth factor receptor, EGFR）基因突变阴性和间变性淋巴瘤激酶（anaplastic lymphoma kinase, ALK）阴性的局部晚期或转移性NSCLC，程序性死亡受体1（programmed cell death 1, PD-1）/程序性死亡配体1（programmed cell death ligand 1, PD-L1）抑制剂联合或不联合化疗均已经成为一线标准治疗方案。帕博利珠单抗（Pembrolizumab, PEM）联合放化疗治疗晚期NSCLC的临床疗效显著，可改善患者的免疫功能，提高生活质量，且不增加不良反应^[4⇓-6]^。现《2023 CSCO非小细胞诊疗指南》将其一线治疗作为I级推荐，其中PD-L1肿瘤细胞阳性比例分数（tumor proportion score, TPS）≥50%为1A类证据，PD-L1 TPS 1%-49%为2A类证据。PEM是一种单克隆抗体，作为PD-1抑制剂可以有效结合PD-1并解除PD-1对T细胞的抑制作用从而杀伤肿瘤细胞^[7]^。KEYNOTE-024研究^[8]^纳入305例PD-L1 TPS≥50%且EGFR/ALK野生型NSCLC（包括腺癌和鳞癌）患者，PEM显著延长无进展生存期（progression-free survival, PFS）[危险比（hazard ratio, HR）=0.50, P<0.001]和总生存期（overall survival, OS）（HR=0.60, P=0.005），且不良事件（adverse events, AEs）发生率低于化疗组，从而发挥抗肿瘤的作用。PEM自2018年在我国上市以来其有效性和安全性便引起广泛关注，但大多研究均基于随机对照试验（randomized controlled trial, RCT）研究，这些试验的严格控制条件与实际临床应用之间存在较大差异。目前针对PEM在治疗晚期NSCLC的中国人群中的真实世界研究（real world study, RWS）数据较少^[9]^。因此，本研究采用回顾性研究设计，基于RWS数据，采用倾向性评分匹配方法减少组间差异，对比PEM联合或不联合化疗与单纯化疗治疗晚期NSCLC患者的临床疗效和安全性，填补PEM在中国晚期NSCLC患者应用的数据空白。相比RCT研究，RWS更能反映PEM在日常临床中的实际应用效果和安全性。

## 1 资料和方法

### 1.1 研究对象

纳入标准包括：（1）根据美国癌症联合会（American Joint Committee on Cancer, AJCC）第八版肺癌肿瘤原发灶-淋巴结-转移（tumor-node-metastasis, TNM）分期为IIIB-IV期；（2）经病理组织诊断为鳞癌或非鳞癌的NSCLC；（3）具有至少1个可测量病灶；（4）干预措施：PEM组接受PEM单药或PEM联合化疗，对照措施为单纯化疗；（5）有详细诊疗记录及影像资料。排除标准包括：（1）合并其他恶性肿瘤；（2）未定期复诊者；（3）仅用药1个周期者；（4）病历及影像资料不全者。

### 1.2 数据收集

RWS数据主要来自中山大学肿瘤防治中心。研究数据从医院His系统中提取，收集患者基线临床病理特征，包括患者入院登记数据（包括姓名、年龄、性别、身高、体重等）、电子病历、处方信息等。安全性评价数据主要来自病史记录，并通过随访进一步补充完善相关信息，对AEs的出现和持续时间、临床表现及严重程度、处理过程和结果进行记录，并让临床负责医师对PEM相关的副作用进行判定。患者治疗结束后，此阶段数据收集主要进行电话随访并保存通话记录，记录患者停用治疗药物的时间、患者后续其他治疗的情况以及疾病进展或死亡时间。该研究经中山大学肿瘤防治中心伦理委员会审批通过（伦理批件号：B2022-153-01）。

### 1.3 观察指标

肿瘤反应根据2017年发布的实体瘤免疫疗效评价标准（modified RECIST 1.1 for immune based therapeutics, iRECIST）^[10]^进行评估，包括完全缓解（complete response, CR）、部分缓解（partial response, PR）、疾病稳定（stable disease, SD）和疾病进展（progressive disease, PD）。主要结果包括PFS和OS。PFS从治疗开始计算，直到PD或因任何原因死亡。OS是从治疗开始日期开始估计，直到因任何原因死亡或最后一次随访的日期（2023年3月31日）。次要结果包括客观缓解率（objective response rate, ORR）[（CR+PR）/（CR+PR+SD+PD）×100%]、疾病控制率（disease control rate, DCR）[（CR+PR+SD）/（CR+PR+SD+PD）×100%]和AEs。以任意等级的AEs发生率、≥3级AEs发生率及免疫相关性AEs发生率作为安全性评价指标。

### 1.4 统计分析

统计分析使用SPSS软件25.0版，采用GraphPad Prism 9.0版绘制图形。采用描述性统计方法分析病例的临床特点。对于符合正态分布的计量资料采用均数±标准差的方式描述数据，采用t检验进行组间比较；对于不符合正态分布的计量资料采用中位数（第25位百分数、第75位百分数）表示，采用Mann-Whitney U检验进行组间比较。计数资料采用卡方检验和Fisher精确概率法（Monte Carlo, MC）进行分析。倾向性评分匹配（propensity score matching, PSM）被用来消除PEM组和化疗组之间的混杂偏差。采用Kaplan-Meier方法评估PFS和OS，Log-rank检验进行组间比较。通过单变量和多变量分析研究预后因素，单变量分析中P<0.20的变量被纳入多变量分析中，用Cox比例风险回归法探讨临床病理特征与PFS或OS之间的关联，数据显示为HR和95%置信区间（confidence interval, CI）。P<0.05被认为差异具有统计学意义。

## 2 研究结果

### 2.1 病例纳入情况

本课题回顾性分析2020年1月至2021年12月在中山大学肿瘤防治中心使用PEM和化疗的630例患者资料，其中PEM组169例，化疗组461例。以PEM组为基础做1:3最近邻匹配，卡钳值设定为0.02。经匹配后共筛选450例患者，其中PEM组149例，化疗组301例。

### 2.2 患者基本信息和临床特点

进行PSM前，PEM组169例，化疗组461例，两组用药方案患者的基线数据显示PEM组与化疗组患者在性别、TNM分期、病理组织学类型、治疗线数（一线、二线及以上治疗）、吸烟情况方面的差异具有统计学意义（P<0.05）。经PSM后，PEM组年龄中位数是60.0（52.0-67.0）岁，BMI中位数是22.8（21.2-25.2）kg/m^2^；化疗组年龄中位数是61.0（55.0-66.5）岁，BMI中位数是22.9（20.8-25.1）kg/m^2^，PSM后和匹配前均无统计学差异（P>0.05）。两组数据匹配前后的基线数据资料见表1。

**表1:** 倾向性评分匹配法配比前后人群主要特征的分布

Characteristics	Before PSM (n=630)				After PSM (n=450)		
	PEM group (n=169)	Chemo group (n=461)	P		PEM group (n=149)	Chemo group (n=301)	P
Gender			0.008				0.999
Male	134 (79.30%)	316 (68.50%)			117 (78.50%)	228 (75.70%)	
Female	35 (20.70%)	145 (31.50%)			32 (21.50%)	73 (24.30%)	
Age (yr), Median (Interquartile)	61.0 (53.0-67.0)	60.0 (53.0-66.0)	0.325		60.0 (52.0-67.0)	61.0 (55.0-66.5)	0.492
BMI, Median (Interquartile)	22.8 (20.6-25.2)	23.1 (20.8-25.1)	0.703		22.8 (21.2-25.2)	22.9 (20.8-25.1)	0.345
TNM			0.049				0.319
III	29 (17.10%)	109 (23.60%)			24 (16.10%)	35 (11.60%)	
IV	125 (74.00%)	330 (71.60%)			112 (75.20%)	250 (83.10%)	
NA	15 (8.90%)	22 (4.80%)			13 (8.70%)	16 (5.30%)	
Pathological histology			<0.001				0.625
Squamous cell carcinoma	52 (30.80%)	60 (13.00%)			39 (26.20%)	53 (17.60%)	
Non-squamous cell carcinoma	116 (68.60%)	396 (85.90%)			110 (73.80%)	248 (82.40%)	
NA	1 (0.60%)	5 (1.10%)					
ECOG PS			0.817				0.455
0-1	141 (83.40%)	394 (85.50%)			125 (83.90%)	257 (85.40%)	
≥2	6 (3.60%)	14 (3.00%)			6 (4.00%)	7 (2.30%)	
NA	22 (13.00%)	53 (11.50%)			18 (12.10%)	37 (12.30%)	
Number of treatment lines			0.038				0.096
First-line	112 (66.30%)	344 (74.60%)			96 (64.40%)	203 (67.40%)	
Second or more line	57 (33.70%)	117 (25.40%)			53 (35.60%)	98 (32.60%)	
Smoking history			<0.001				0.115
Previous	89 (52.70%)	186 (40.30%)			80 (53.70%)	153 (50.80%)	
Never	62 (36.70%)	252 (54.70%)			61 (40.90%)	129 (42.90%)	
NA	18 (10.60%)	23 (5.00%)			8 (5.40%)	19 (6.30%)	

PEM: Pembrolizumab; Chemo: chemotherapy; BMI: body mass index; TNM: tumor node metastasis; NA: not available; ECOG: Eastern Cooperative Oncology Group; PS: performance status; PSM: propensity score matching.

### 2.3 有效性评价结果

患者使用PEM的疗效以ORR、DCR、PFS和OS进行判断。PSM后的450例患者中有446例患者进行了疗效评估，其中PEM组有146例患者，ORR和DCR分别为48.63%和95.21%；化疗组有300例患者，ORR和DCR分别为36.00%和89.67%。两组的ORR差异具有统计学意义（P=0.011），DCR无统计学差异（P=0.069）（表2）。

**表2:** 最佳总体疗效

Characteristics	CR	PR	SD	PD	ORR	DCR
Best overall efficacy after PSM
PEM group (n=146)	0 (0.00%)	71 (48.63%)	68 (46.57%)	7 (4.80%)	71 (48.63%)	139 (95.21%)
Chemo group (n=300)	1 (0.33%)	107 (35.67%)	161 (53.67%)	31 (10.33%)	108 (36.00%)	269 (89.68%)
P					0.011	0.069
Best overall efficacy of first-line therapy after PSM
PEM group (n=95)	0 (0.00%)	55 (57.89%)	38 (40.00%)	2 (2.10%)	55 (57.89%)	93 (97.89%)
Chemo group (n=202)	1 (0.50%)	73 (36.14%)	110 (54.45%)	18 (8.91%)	74 (36.63%)	184 (91.09%)
P					0.001	0.029
Best overall efficacy of second and more lines therapy after PSM
PEM group (n=51)	0 (0.00%)	16 (31.37%)	30 (58.82%)	5 (9.80%)	16 (31.37%)	46 (90.20%)
Chemo group (n=98)	0 (0.00%)	34 (34.69%)	51 (52.04%)	13 (13.26%)	34 (34.69%)	85 (86.73%)
P					0.684	0.538

CR: complete response; PR: partial response; SD: stable disease; PD: progressive disease; ORR: objective response rate; DCR: disease control rate.

根据收集到的数据进一步筛选匹配后一线使用免疫治疗和化疗的患者进行最佳总体疗效评价。一线PEM组排除疗效未知的患者后共有95例患者入组，一线化疗组排除疗效未知的患者后共有202例患者入组。结果显示PEM组和化疗组的ORR和DCR的差异均具有统计学意义（P<0.05），匹配后PEM组和化疗组ORR分别为57.89%和36.63%，DCR分别为97.89%和91.09%，PEM组的ORR和DCR均高于化疗组（表2）。二线及以上PEM组排除疗效未知的患者后共有51例患者入组，化疗组共有98例患者入组，结果显示PEM组和化疗组的ORR和DCR的差异无统计学意义（P>0.05）（表2）。

到最后一次随访时，纳入的全部患者中有281例患者（63.0%）出现了PD，其中PEM组有91例患者（62.33%），化疗组有190例患者（63.3%）。PEM组和化疗组患者的中位PFS分别为15.5个月（95%CI: 11.8-19.2）和8.8个月（95%CI: 7.5-10.1），HR为0.611（95%CI: 0.483-0.774，P<0.001，图1A）。PEM组和化疗组患者的中位OS分别为未达到和26.2个月（95%CI: 22.1-30.3），HR为0.532（95%CI: 0.399-0.709，P<0.001，图2A）。在匹配后的一线患者中，PEM组和化疗组患者的中位PFS分别为15.5个月（95%CI: 12.4-18.6）和9.6个月（95%CI: 7.5-11.7），HR为0.615（95%CI: 0.458-0.824，P=0.002，图1B）；PEM组和化疗组患者的中位OS分别为未达到和27.7个月（95%CI: 20.2-35.2），HR为0.538（95%CI: 0.378-0.765，P=0.002，图2B）。在匹配后的非鳞癌患者中PEM组和化疗组患者的中位PFS分别为17.4个月（95%CI: 15.0-19.8）和8.8个月（95%CI: 7.0-10.6），HR为0.582（95%CI: 0.446-0.760，P<0.001，图1C）；中位OS分别为未达到和27.0个月（95%CI: 17.1-36.9），HR为0.476（95%CI: 0.330-0.688，P<0.001，图2C）。在匹配后的鳞癌患者中PEM组和化疗组患者的中位PFS分别为10.0个月（95%CI: 4.2-15.8）和8.0个月（95%CI: 5.1-10.9），HR为0.658（95%CI: 0.392-1.108，P=0.100，图1D）；中位OS分别为30.5个月（95%CI: NR）和27.7个月（95%CI: 16.7-38.7），HR为0.726（95%CI: 0.367-1.440，P=0.335，图2D）。在匹配后的二线及后线治疗人群中PEM组和化疗组的中位PFS分别为11.7个月（95%CI: 4.7-18.7）和7.7个月（95%CI: 7.0-8.4），HR为0.600（95%CI: 0.404-0.893，P=0.013，图1E）；中位OS分别为未达到和25.6个月（95%CI: 22.1-29.1），HR为0.526（95%CI: 0.319-0.866，P=0.025，图2E）。

**图1:**
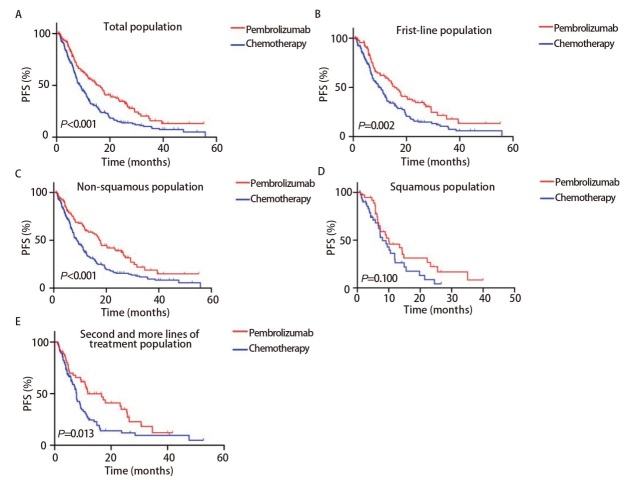
比较帕博利珠单抗组和化疗组的PFS的Kaplan-Meier曲线。A：总人群；B：一线治疗人群；C：非鳞状细胞癌人群；D：鳞状细胞癌人群；E：二线及以上人群。

**图2:**
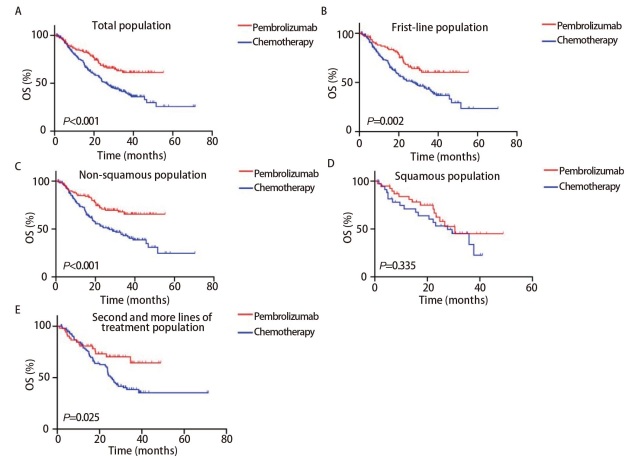
比较帕博利珠单抗组和化疗组的OS的Kaplan-Meier曲线。A：总人群；B：一线治疗人群；C：非鳞状细胞癌人群；D：鳞状细胞癌人群；E：二线及以上人群。

进一步分析PFS与基线因素之间的关系（表3），将单因素分析中P<0.2的因素纳入多因素分析（治疗线数和病理组织学类型），结果显示没有与PFS有关联的因素。同时，对OS与基线因素之间的关系进行研究，结果显示（表3）在单因素分析中P<0.2的因素只有TNM分期，进行多因素分析的结果显示TNM分期与OS无明显关联。

**表3:** 总人群中的PFS和OS单因素和多因素分析

Characteristics	n*	Categorization	PFS					OS			
			Univariable HR (95%CI)	Univariable P	Multivariable HR (95%CI)	Multivariable P		Univariable HR (95%CI)	Univariable P	Multivariable HR (95%CI)	Multivariable P
Age (yr)	450	≥65 vs <65	1.097 (0.857-1.404)	0.453	-	-		1.134 (0.845-1.521)	0.392	-	-
Gender	450	Female vs Male	1.101 (0.834-1.454)	0.505	-	-		0.984 (0.715-1.353)	0.919	-	-
Smoking history	423	Yes vs No	0.939 (0.738-1.196)	0.609	-	-		0.904 (0.682-1.200)	0.483	-	-
Histology	450	Non-squamous cell carcinoma vs Squamous carcinoma	0.810 (0.595-1.102)	0.148	0.802 (0.599-1.073)	0.137		0.835 (0.585-1.192)	0.293	-	-
ECOG	395	≥2 vs 0-1	1.320 (0.619-2.817)	0.409	-	-		1.323 (0.523-3.348)	0.498	-	-
TNM	421	IV vs III	0.806 (0.548-1.185)	0.228	-	-		0.745 (0.476-1.165)	0.148	1.344 (0.898-2.012)	0.150
Number of treatment lines	450	First line vs Second or more line	0.836 (0.649-1.076)	0.149	1.205 (0.942-1.541)	0.137		1.010 (0.754-1.352)	0.948	-	-

*There were 27 patients with no smoking history in their medical records, 55 patients with no ECOG score in their medical records, and 29 patients with no TNM stage in their medical records.

### 2.4 安全性评价结果

RWS结果显示在PSM后的450例患者中，出现AEs的患者在PEM组中有119例（79.87%），化疗组有261例（86.71%），两组之间没有统计学差异（P=0.059）。≥3级AEs两组分别有6例（4.03%）和22例（7.31%）患者。PEM组中最常见的AEs是贫血（52.35%）、咳嗽（20.80%）和白细胞计数减少（19.46%）。化疗组中最常见的AEs是贫血（69.77%）和白细胞计数减少（20.60%）。另外在病历记录中还发现PEM组有2例I-II度的免疫相关性肺炎患者。详见表4。没有5级AEs。

**表4:** 两组药物不良反应

AEs	PEM group			Chemotherapy group		P (Grade≥3)
	Any grade	Grade≥3		Any grade	Grade≥3	
Any AE	119 (79.87%)	6 (4.03%)		261 (86.71%)	22 (7.31%)	0.175
Anemia	78 (52.35%)	3 (2.01%)		210 (69.77%)	8 (2.66%)	>0.999
Neutropenia	28 (18.79%)	1 (0.67%)		41 (13.62%)	9 (2.99%)	0.176
Leukopenia	29 (19.46%)	0 (0.00%)		62 (20.60%)	4 (1.33%)	0.307
Thrombocytopenia	14 (9.40%)	2 (1.34%)		28 (9.30%)	2 (0.66%)	0.602
Creatinine elevation	12 (8.05%)	0 (0.00%)		25 (8.31%)	0 (0.00%)	-
AST elevation	11 (7.38%)	0 (0.00%)		12 (3.99%)	0 (0.00%)	-
ALT elevation	21 (14.09%)	0 (0.00%)		15 (4.98%)	2 (0.66%)	>0.999
Hypothyroidism	8 (5.37%)	0 (0.00%)		0 (0.00%)	0 (0.00%)	-
Hyperthyroidism	1 (0.67%)	0 (0.00%)		0 (0.00%)	0 (0.00%)	-
Rash	13 (8.72%)	0 (0.00%)		4 (1.33%)	0 (0.00%)	-
Pruritus	10 (6.71%)	0 (0.00%)		4 (1.33%)	0 (0.00%)	-
Decreased appetite	8 (5.40%)	0 (0.00%)		39 (12.96%)	0 (0.00%)	-
Fatigue	24 (16.11%)	0 (0.00%)		29 (9.63%)	0 (0.00%)	-
Cough	31 (20.80%)	0 (0.00%)		46 (15.28%)	0 (0.00%)	-
Fever	9 (6.04%)	1 (0.67%)		12 (3.99%)	0 (0.00%)	0.331
Constipation	7 (4.70%)	0 (0.00%)		12 (3.99%)	0 (0.00%)	-
Headaches	9 (6.04%)	0 (0.00%)		12 (3.99%)	1 (0.33%)	>0.999
Vomiting	9 (6.04%)	0 (0.00%)		23 (7.64%)	0 (0.00%)	-
Edema of the extremities	4 (2.68%)	0 (0.00%)		7 (2.33%)	0 (0.00%)	-
Dyspnea	4 (2.68%)	0 (0.00%)		15 (4.98%)	1 (0.33%)	>0.999
Nausea	12 (8.05%)	0 (0.00%)		41 (13.62%)	1 (0.33%)	>0.999
Diarrhea	5 (3.36%)	0 (0.00%)		11 (3.65%)	0 (0.00%)	-
Pneumonitis	0 (0.00%)	0 (0.00%)		3 (1.00%)	0 (0.00%)	-
Immune-related pneumonia	2 (1.34%)	0 (0.00%)		0 (0.00%)	0 (0.00%)	-

AEs: adverse events; AST: aspartate aminotransferase; ALT: alanine aminotransferase.

## 3 讨论

过去NSCLC的抗肿瘤治疗通常采用以铂类药物为基础的化疗作为首选方案。随着分子靶向药物的开发，携带驱动基因突变患者的治疗成效得到了显著提升。尽管如此，对于无突变患者有效的治疗选择仍然较少^[11,12]^。PD-1/PD-L1抑制剂通过阻断PD-1与其配体的结合可以抑制肿瘤细胞的免疫逃逸，从而发挥其抗肿瘤效果。目前，这类抑制剂已被广泛用于治疗晚期NSCLC患者。在临床实践中，如何从众多治疗方案中选择最佳治疗方案显得尤为重要^[13⇓-15]^。

在本研究中，接受PEM组治疗的晚期NSCLC患者的ORR为48.63%，观察到的ORR与KEYNOTE-024^[8]^（44.8%）和KEYNOTE-189^[16]^（47.6%）试验相似。KEYNOTE-024试验中PEM单药治疗PD-L1高表达（≥50%）的NSCLC患者的中位PFS为10.3个月，中位OS未达到，KEYNOTE-189试验中PEM联合化疗的中位PFS和中位OS分别为8.8和22.0个月，本研究结果显示，PEM组的中位PFS和中位OS分别为15.5个月和未达到。尽管RWS数据和临床试验数据存在差异，但本研究中的RWS数据结果与既往临床试验结果在趋势上基本一致，进一步验证了PEM在真实世界中的有效性和安全性。这一结果对临床实践具有重要指导意义，因为它展示了PEM在多样化患者群体中的应用效果。一项基于日本人群的回顾性研究^[17]^报道PD-L1≥50%的NSCLC患者一线使用PEM单药治疗的中位PFS为18.4个月，中位OS未达到。一项基于中国人群的回顾性研究^[18]^报道NSCLC患者一线使用PEM联合化疗治疗的中位PFS和OS分别为10.0个月和未达到。这些研究结果与本次研究结果相似。另外，本次研究中化疗患者的OS比先前已发表研究要长，可能的原因是化疗组的部分患者在后线治疗中交叉使用了免疫治疗药物，如信迪利单抗、卡瑞利珠单抗、特瑞普利单抗、替雷利珠单抗或纳武利尤单抗。

此外，本研究进一步探索了PEM组和化疗组在一线和二线及以上患者人群中的疗效，结果表明在一线患者人群中PEM组的ORR、PFS和OS均优于化疗组。分析这一结果的原因可能是化疗可通过多种免疫调节机制增强机体的免疫功能，如减少免疫抑制细胞的数量和活性、诱导免疫原性细胞凋亡、增强肿瘤抗原的呈递、促进树突状细胞的成熟以及调节效应T细胞的功能，从而实现对肿瘤细胞的有效杀伤。此外，化疗可诱导肿瘤细胞PD-L1表达，与表面PD-1（CD28免疫球蛋白超家族成员）结合，形成抑制T细胞活性的通路，是肿瘤免疫逃逸的关键机制之一。PD-1/PD-L1抑制剂通过阻断此通路，可重塑患者免疫系统，增强其抗肿瘤能力^[19⇓⇓-22]^。值得注意的是，本研究结果表明在二线及以上患者人群中PEM组的PFS和OS优于化疗组，结果具有统计学意义。这一结果与一项已发表的回顾性研究^[23]^结果相似，该研究回顾性分析了北京协和医院连续接受免疫检查点抑制剂（immune checkpoint inhibitors, ICIs）（纳武利尤单抗或帕姆单抗）单药治疗的患者，该研究表明ICIs在二线及以上NSCLC治疗中的实际临床结果是有希望的。除此之外，本研究还发现在鳞癌患者人群中PEM组的PFS和OS与化疗组没有统计学差异，这可能是由于纳入鳞癌患者样本量相对较少，且进行患者随访时有部分患者手机号注销或停机，无法确认患者的生存状态而对这部分患者的数据进行了删失处理所导致。由此可见，PEM在一线人群的ORR、PFS和OS均优于化疗，在二线及以上NSCLC中也显示出良好的临床结果。但是本研究也有一定的局限性：（1）代表性不足和随访时间不够长，由于本研究的数据来源于单一的三级甲等医院，可能无法全面代表更广泛的患者人群，不同医院和地区的患者特征、治疗习惯和医疗水平可能存在差异，这可能会影响结果的普遍性；（2）回顾性研究不可避免地存在选择偏倚，虽然研究采用了PSM来减少组间差异，但仍然无法完全消除未测量的混杂因素的影响；（3）研究中接受PEM治疗的患者可能在治疗方案上存在一定的异质性，包括单药和联合化疗的不同组合，这可能会影响结果的解释；（4）未考虑部分患者的基因突变状态，回顾性研究依赖于现有的医疗记录和随访信息，可能存在记录不全或信息遗漏的情况，这可能导致信息偏倚。

综上，与单纯化疗相比，PEM联合或不联合化疗更具有优势，可以获得更好的治疗效果且不增加AEs，深入的研究还需要根据PD-L1的表达水平和EGFR/ALK基因状态等因素进行进一步分析，将来在数据可获得的情况下可得到更为科学严谨的安全性和有效性的评价结果。
